# Transient liver elastography in the follow-up of Fontan patients: results of a nation wide survey in Germany

**DOI:** 10.3389/fped.2023.1194641

**Published:** 2023-08-30

**Authors:** Zora Meyer, Nikolaus Haas, Richard Mühlberg, Annabell Braun, Markus Fischer, Guido Mandilaras

**Affiliations:** ^1^ Ludwig Maximilian University of Munich, Munich, Germany; ^2^ LMU Munich University Hospital, Munich, Germany

**Keywords:** Fontan, liver, elaslography, fibrosis, patient

## Abstract

**Introduction:**

Fontan-palliated patients are at risk for the development of Fontan-associated liver disease (FALD). Currently, there is no consensus on how to stage FALD. Transient elastography (TE) is a rapid, non-invasive method to assess FALD and liver fibrosis.

**Method:**

To assess the availability and conditions of using TE to monitor liver disease in Fontan patients in german centers for pediatric cardiology and to propose the introduction of a standardized national protocol for the monitoring of liver disease, we developed a questionnaire.

**Results:**

In total, 95 valid questionnaires were collected. Only 20% of the centers offer the TE investigation directly. Most of the centers transfer the patients to another department or center (40%) or didńt offer TE (40%). In only 2.6% of the centers TE is performed directly by the cardiologist. Most of the centers transfer the patients to a other department. In 29.2% TE is performed only at a certain age of the patients and in 27.7% it is performed if the patients present symptoms of failing Fontan. In only 13.9% of the centers TE is proposed in all the Fontan patients on a routine basis. Most often TE is performed only from the beginning of the adolescence. In the majority of answers it was not known if the patients are fasting for the examination (68%) or not and if the TE examination had to be performed in a specific breathing phase during TE (Inspiration/Expiration, 90%). In the majority, TE is not offered routinely (46.9%).

**Discussion:**

To date in Germany, TE is only used in a few numbers of centers specialized in Fontan follow-up. A standardized protocol to use TE is currently not existing. With regard to the feasibility of the examination, it is evident that TE is a quick, cheap and easy method to distinguish between cases with and without progressive FALD. This makes TE a useful and prognostic tool for screening of liver disease and to failing Fontan circulation.

**Conclusion:**

We propose a systematic TE evaluation of possible liver congestion and fibrosis, as a part of the routine follow-up of Fontan patients.

## 1. Introduction

The Fontan procedure was introduced in 1968 as long-term palliation for patients with tricuspid atresia (1). This procedure creates artificial circulation with two serial capillary beds by connecting the caval veins to the pulmonary artery (1). The procedure has evolved to become the surgical strategy for all congenital heart lesions with an anatomical or functional single ventricle. Today, a large number of these patients reach adulthood (2, 3). Protein-losing enteropathy, plastic bronchitis, arrhythmias, and Fontan-associated liver disease (FALD) are long-term complications after Fontan palliation (4). For pediatric patients, the prevalence and impact of liver disease are less well explored (5, 6). Hemodynamic changes associated with the Fontan circulation (including a chronic increase of the central venous pressure and decrease of cardiac output after Fontan surgery) contribute to the development of Fontan-associated liver disease (FALD) (7, 8). Fontan circulation creates the condition for severe venous hepatic stasis, with central vein and sinusoid dilatation. These changes may lead to chronic liver injury, fibrosis liver cirrhosis, and even hepatocellular carcinoma (HCC) (9). The prevalence of FALD is not well defined as routine evaluation of the liver has not yet become standard care for these patients. Currently, there is no consensus on how to stage FALD. The liver biopsy is a highly valued diagnostic procedure and for a long time was considered to be the “Gold standard” method for grading the degree of liver fibrosis. It is applicable in the Fontan cohort with a reasonable morbidity and a low mortality risk. These risks result mainly from the high venous pressure and the use of anticoagulation therapy in these patients (10). Noninvasive methods for monitoring liver fibrosis are under development; standards of use are however lacking. Noninvasive diagnostic methods can identify changes in the liver parenchyma. Transient elastography (TE) has emerged as a valuable tool for evaluating liver fibrosis in a variety of diseases including viral hepatitis and nonalcoholic fatty liver disease (11). TE is able to assess liver fibrosis by measuring liver stiffness. The transducer transmitted vibrations of mild amplitude and low frequency (50 Hz) to the tissue, inducing an elastic shear wave that propagates through the tissue (12). Pulse-echo ultrasound is used to follow the propagation of the shear wave and measure its velocity, which is related to tissue stiffness: the stiffer the tissue, the faster the shear wave propagates (13). Liver Stiffness values range from 1.5 to 75 kPa, and lower values indicate a more elastic liver (14). TE was validated in children with liver fibrosis receiving liver biopsy (15), and the evaluation by TE proved to be in agreement with histopathologic findings in Fontan patients (16). In general, the TE can provide a promising novel non-invasive method for long-term follow-up of patients with Fontan circulation; this may be especially important for individual changes during routine follow-up. By TE, we are able to assess and monitor Fontan-associated liver disease even though further studies for the standardization of TE are needed (17). Unfortunately, a universally accepted follow-up imaging protocol for the liver has not been established. Our study aimed to assess if and how TE is practiced in the follow-up of Fontan patients in those German pediatric cardiology centers caring for Fontan patients. We report here the results and analysis of a national questionnaire, and we will propose a systematic TE evaluation of possible liver congestion and fibrosis, as a part of the routine follow-up of Fontan patients.

## 2. Materials and methods

This study focuses on a national German multi-center survey to assess the practice of TE in the follow-up of Fontan patients through the use of a questionnaire.

### 2.1. Questionnaire

We have created a short questionnaire made up of 10 items to assess the use of TE in German centers and hospitals for pediatric cardiology. The 10 items have been categorized into three parts:

I. General information on whether the TE is practiced and how it is done
II. Indication for TE
III. Condition of the patient to practice TE

The details of the questionnaire are shown in Table 1. Part I consists of the following five items:

A. Has the TE been proposed in the center? Four options were pre-proposed: yes/no, offer to transfer the patient to another department, or offer to transfer the patient to another center.
B. Who is performing TE? The pediatric cardiologist, the pediatric department, the pediatric radiologic department, the adult radiologic department, or it is not known who is performing it.
C. Which tool is used? Philips, GE, Siemens, Toshiba, or it is not known what tool is being used.
D. TE has been offered for how long? Seven options were proposed: it has been offered for less than a year, for 1–2 years, for 3–5 years, for 5–10 years, it is currently in planning, it is not offered routinely, or it is not known for how long it has been offered.
E. How many TEs are performed annually? Six options were proposed: fewer than 50, 50–100, 100–200, more than 200, it is not proposed, or it is not known how many times it is proposed.

**Table 1:** Questionnaire.

1. Has the TE been proposed in the center?	2. Who is performing the TE?	3. Which tool is used?	4. TE has been offered for how long?	5. How many TE are performed annually?
Yes	The pediatric cardiology	Philipps	<1 year	<50
No	The pediatric department	GE	1–2 years	50–100
In a other department	The Adult radiologic department	Simens	3–5 years	100–200
In a other center	The pediatric radiologic department	Toshiba	5–10 years	>200
	It is unknown	It is unknown	It is in planing	It isńt proposed
			It isńt offered	It is unknown
6. Which is the indication to perform a TE?	7. If TE is proposed, at which age TE it is performed?	8. At which interval TE is offered?	9. Was the patient fasting for TE examination?	10. In which breathing phase TE was performed?
All patient independant from age	3–5 years	If symptoms	Yes	Inspiration
All patient from beginning of certain age	At school age	1/year	No	Expiration
Only by presenting symptoms	Before adolescence	1–2 years	It is irrelevant	It is irrelevant
Other indications	>16 years	3–5 years	It is unkown	It is unknown
	>18 years	5–10years		
	Only if indicated	Other/unknown		

Part II consisted of the following three items:

A. What is the indication to perform at the center TE on Fontan patients? Four options were proposed: on all patients independent of their age, on all patients starting at a certain age, only if the patients present symptoms of failing Fontan, or because of other indications.
B. If TE is proposed, at what age is it performed? Seven options were proposed: when reaching 3–5 years old, when reaching school age (6 years old), before the beginning of adolescence (10 years old), starting at the beginning of adolescence, not before the age of 16 years old, not before the age of 18 years old, or only if indicated, independent of the age of the patient.
C. At which interval is TE offered? Only if symptoms appear (failing Fontan, ascites), once a year, at an interval of 1–2 years, at an interval of 3–5 years, at an interval of 5–10 years, or other/unknown.

Part III consisted of two items:

A. Was the patient fasting for the examination? The options were as follows: yes, no, it is irrelevant, or it is not known,
B. In which breathing phase is TE performed? The options were as follows: inspiration, expiration, it is irrelevant, or it is not known.

### 2.2. Hospitals

In Germany, we have 35 university hospitals and dedicated heart centers that perform surgery and/or diagnostics as well as interventional catheter investigations in Fontan patients. The follow-up of Fontan patients is however not only performed in these centers but additionally in many tertiary referral pediatric hospitals (without cardiac surgery and/or catheter) by experienced pediatric cardiologists. In total the number is about 100. A total of 95 German hospitals were included in the study. After having made initial personal contact, we sent an e-mail to inform the participating doctors of the study. Afterward, an online questionnaire was sent to the participants via e-mail, using the internet-based surveying tool called “Surveymonkey” (Version 2021). All data was collected. Statistical analysis was performed subsequently using descriptive statistics.

## 3. Results

In total, the questionnaires were sent to 35 cardiac centers and 100 referral hospitals which are caring for patients after the Fontan operation. As a result, 95 valid questionnaires were collected, which is equivalent to a response rate of around 70%.

### 3.1. TE examination

The results of this part of the questionnaire are shown in Table 2. According to the results gathered from the questionnaires, only 20% of the cardiac hospitals directly offer TE during cardiac follow-up examinations, and 40% of the hospitals transfer the patients to another department (i.e., radiology and gastroenterology) or another center. In addition, 40% of the hospitals do not offer TE or see an indication for TE.

**Table 2:** TE examination.

1. Has the TE been proposed in the center?		2. Who is performing the TE?		3. Which tool is used?		4. TE has been offered for how long?		5. How many TE are performed annually?	
Yes	20%	Pediatric cardiology	2.6%	Philips	10%	<1 year	1.5%	<50	23.3%
No	40%	Pediatric department	7.9%	GE	8%	1–2 years	7.8%	50–100	10%
Other department	20%	Adult radiologic department	7.9%	Siemens	6%	3–5 years	21.9%	100–200	1.7%
Other center	20%	Pediatric radiologic department	23.5%	Toshiba	0%	5–10 years	9.4%	>200	0%
		Unknown	57.8%	Unknown	78%	In planning	3.2%	It isńt proposed	38.5%
						isńt offered	46.9%	Unknown	26.5%
						Unknown	9.3%		
** **	**100%**		**100%**	** **	**100%**		**100%**		**100%**

In only 2.6% of the hospitals, TE is performed directly by the cardiologist. Of the hospitals, 23.5% transfer the patients to a pediatric radiologic department, 7.9% to an adult radiologic department, and 7.9% to another pediatric center. For 57.8% of the hospitals, it is not known whether TE is performed and who is performing it.

TE is performed with either the Philipps ultrasound equipment (10%), with GE (8%), or with Siemens (6%), and in 72% of the cases, it is not known what the type of equipment used is.

In 9.4% of the hospitals, TE has been offered for 5–10 years. In 21.9% of the hospitals, it has been offered for 3–5 years. In 7.8% of the hospitals, it has been offered for 1–2 years. In 1.5% of the hospitals, it has been offered for less than a year. In 3.2% of the hospitals, it is currently being planned for the future. In the majority of the hospitals (46.9%) TE is not offered routinely, and in 9.3% of the hospitals, it is not known when TE was first offered.

Of the hospitals, 23.3% perform fewer than 50 TE per year, 10% perform 50–100 TE per year, 1.7% perform 100–200 TE per year, 38.3% do not see an indication, and for 26.5% of the hospitals, the number of performed TEs is not known (see Table 1).

### 3.2. Patient indication

The results of this part of the questionnaire are shown in Table 3. When TE was indicated and used on Fontan patients, in 29.2% of the hospitals, TE is only performed when patients have reached a certain age, and in 27.7% of the hospitals, it is only performed if the patients present symptoms of failing Fontan. In only 13.9% of the hospitals is TE performed on all Fontan patients. In 29.1% of the hospitals, TE is performed for other indications, such as deterioration.

**Table 3:** TE indication.

1. Which is the indication to perform a TE?		2. If TE is proposed, at which age TE it is performed?		3. At which interval TE is offered?	
All patients independant from age	13.9%	3–5 years	0%	If symptoms	35.3%
All patient sfrom beginning of certain age	29.2%	At school age	12.5%	1/year	11.6%
Only by presenting symptoms	27.7%	Before adolescence	15.6%	1–2 years	31.6%
Other indication	29.2%	From beginning of adolescence	28.1%	3–5 years	3.3%
		>16 years	3.1%	5–10 years	0%
		>18 years	6.2%	Other/unknown	18.2%
		Only if indicated	34.5%		
	**100%**		**100%**		**100%**

In the hospitals where TE is routinely performed we found the following: for 28.1% of the hospitals, it is only performed starting at the age of adolescence; for 15.6%, before the beginning of adolescence (10 years); for 12.5%, for those already in school age (6 years), for 3.1% not before the age of 16 years; and for 6.2% not before an age of 18 years. Additionally, 34.5% of the hospitals performed TE only for a given indication, independent of the age of the patient.

Furthermore, 31.6% of the TE examinations are performed either once a year or every 2 years; 11.6% of the TE examinations are performed regularly and annually and 3.3% at an interval of 3–5 years; 35.3% of the hospitals performed TE only by indication, independent of the interval of the examination; 18.2% of the centers answered that the interval is not known.

### 3.3. Patient preparation

The results of this part of the questionnaire are shown in Table 4. For 68.7%, it was not known if the patients are fasting for the examination, and for 8.3%, it was deemed irrelevant. For 18% the patient was not fasting, and for only 5% the patient was fasting. In most cases (90%) the breathing phase (Inspiration/Expiration) during TE was not known.

**Table 4:** Patient preparation.

Was the patient fasting for TE examination?		In which breathing phase TE was performed?	
Yes	5%	Inspiration	3.45%
No	18%	Expiration	3.45%
Irrelevant	83.%	Irrelevant	3.45%
Unknown	68.7%	Unknown	89.65%
	**100%**		**100%**

## 4. Discussion

The Fontan operation is a palliative procedure proposed for children with congenital heart lesions with an anatomical or functional single ventricle. Over the last few years, early and long-term survival has improved and many of the patients are reaching adulthood (2, 3). However, this longer survival can lead not only to cardiac complications but also to long-term extra-cardiac complications (18).

### 4.1. Fontan associated liver disease (FALD)

In recent years, Fontan associated liver disease (FALD) has gained increased interest and importance in the long-term outcome after Fontan-surgery (19). Multiple factors, including hypoxia, chronic elevation of the central venous pressure, and a decreased cardiac output after Fontan surgery may all contribute to this variant of congestive hepatopathy, which is characterized by sinusoidal and portal fibrosis (6, 19). The prevalence of FALD is not well defined yet. In recent years, it has become evident that FALD can progress to cirrhosis or hepatocellular carcinoma (20). It has been demonstrated from biopsy data that liver disease is present and progressive in most Fontan patients (21).

### 4.2. Diagnostic approach

To date, there is no consensus on guidelines for diagnosing and monitoring FALD (22). The literature shows that the time post-Fontan is the most important predictor of advanced FALD (23–25). In the literature it is also reported that 10–15 years post Fontan completion, signs of fibrosis appear in the biopsy (20, 23, 24). In a study involving 57 adolescent patients undergoing surveillance cardiac catheterization and liver biopsy, 10 years after Fontan-surgery, Patel et al. reported that 56/57 patients had histologic evidence of fibrosis. A total of 19 patients demonstrated bridging fibrosis or cirrhosis (23). Silva-Sepulvedda et al. also reported in a surveillance cardiac catheterization and liver biopsy cohort involving 49 patients 15.2 years post Fontan that all patients had histologic evidence of fibrosis (24). However, early detection of liver injury is important to assess the risk of advanced liver disease and to evaluate the option of early treatment and intervention. The best methods for surveillance of the hemodynamic status of a Fontan patient and associated liver health are still being investigated and debated.

### 4.3. Transjugular liver biopsy

Some centers have begun surveillance cardiac catheterization combined with transjugular liver biopsy in all patients 10–15 years post Fontan (23, 24). The staging of liver fibrosis in FALD requires a multi-modality approach involving clinical assessment, biochemical/hematological parameters, non-invasive fibrosis scores, radiological imaging, elastography, and liver histology (26, 27). The biopsy is considered to be the “gold standard” procedure to stage fibrosis. Fontan patients have however an increasing risk during conventional percutaneous liver biopsy because of anticoagulation therapy and anatomy (28). In up to 7.4% of cases, hemorrhage is reported in Fontan patients after conventional liver biopsy (29).

### 4.4. Laboratory investigations and scoring

Standard laboratory investigations are of limited value in the staging of liver fibrosis. Liver enzymes are frequently normal, even in advanced disease (26, 30). Certain predictive scores based on serum markers were developed for staging liver cirrhosis across a range of chronic liver diseases. These include the serum aspartate aminotransferase to alanine aminotransferase ratio (AST/ALT ratio), the Aspartate Aminotransferase to platelet ratio index (APRI) score, the Fibrosis-4-scores (FIB), the Forns Index, and the “Model for End-stage Liver disease” (MELD) score (31, 32). In the study from Baek et al., the Forns index was the best predictor of cardiac hepatopathy (32). The APRI (Aspartate Aminotransferase to platelet ratio index) score and the FIB (fibrosis)-4 scores had only modest discriminatory power in identifying patients with advanced fibrosis (31, 33). The value of laboratory examination in identifying progressive, clinically significant liver disease is therefore limited, as the majority of patients will have no detectable abnormalities (31).

### 4.5. Imaging

In patients with congestive hepatopathy, ultrasound (US), computed tomography (CT), and magnetic resonance imaging (MRI) have been used to detect change in the liver (26, 31, 34). US is a valuable tool to identify FALD and may be useful to identify the progression of FALD before biochemical hepatic dysfunction (26, 32). In a study involving 46 adolescent patients undergoing surveillance US and MRI, Thrane et al. reported low sensitivity of US for identifying hepatic nodules compared to MRI. Of the 46 involved patients, 3 have nodules ≥1 cm insize on the US, and 7 of the 46 patients have nodules ≥1 cm in size on the MRI (26). CT and MRI are better able to detect liver masses and liver architecture than US (26, 27).

### 4.6. Elastography

Currently, elastography has emerged as a potentially useful tool to diagnose and monitor liver health in various conditions (11). Elastography can assess liver stiffness, and liver stiffness does increase as fibrosis progresses (35). Recently, three tools have been used to measure liver stiffness: MRI elastography, shear wave elastography, and transient elastography (TE). TE is a noninvasive ultrasound tool that is technically simple and easy to apply, has a high accuracy, and is well accepted by the patient (36, 37). TE assesses liver fibrosis by measuring liver stiffness and shows a high reproducibility. The transducer transmits vibrations of mild amplitude and low frequency (50 Hz) to the tissue, inducing an elastic shear wave that propagates through the tissue (12, 38). Pulse-echo ultrasound is used to follow the propagation of the shear wave and measure its velocity, which is related to tissue stiffness: the stiffer the tissue, the faster the shear wave propagates (13). There are different techniques available depending on the equipment used (i.e., Siemens, Philips, GE, etc.). Even though the absolute values may differ between the ultrasound machines, the long-term assessment with a dedicated team is usually performed with the same equipment, and long-term changes can be detected. The TE has emerged as a valuable tool for evaluating liver fibrosis in a variety of diseases including viral hepatitis and nonalcoholic fatty liver disease (11). Schäfer et al. reported that the results of the TE correlate well with histological scores in 75 children with liver disease (39). Also, Kutty et al. reported that for 41 patients with Fontan circulation, the results by TE proved to be in agreement with the pathologic findings (16). In a study of 46 adult patients who underwent cardiac catheterization and liver elastography, a positive correlation between liver stiffness and the venous pressure in the Fontan circulation was observed (40). Several small studies have suggested that unfavorable hemodynamic cardiac catheterization values and high TE measurements may be associated with high Fontan pressure (16, 24, 25, 27, 40). Wu et al. also demonstrated that high liver stiffness and advanced hepatic fibrosis in the biopsy is associated with unfavorable hemodynamics assessed by cardiac catheterization (41). In summary, TE may be a helpful tool to detect increasing congestive hepatopathy from failing Fontan hemodynamics. It may also be an additional argument to optimize hemodynamics and to reduce pulmonary vascular resistance and pressures. Egbe et al. detected that changes in TE measurements were associated with clinical deterioration. Therefore, sequential TE measurements may be useful for monitoring patients over time (42). Fontan-associated liver disease starts basically immediately after Fontan completion and altered hemodynamics including elevated central venous pressure play a major role. During the early period until puberty, the clinical implications are however low. This changes after puberty according to all literature available on the matter. Usually, Fontan patients are assessed by a pediatric cardiologist once or twice a year—even when no overt problems or complications are observed. In order to assess the individual TE values over time, some initial measurements before puberty are important, and yearly assessment during routine follow-up seems logical because of the possible changes thereafter. LS cutoff values derived from chronic liver disease of other etiologies cannot most likely be directly applied to predict the extent of liver fibrosis in Fontan patients (17). Fontan patients show higher values of liver stiffness than in conventional patients, and the relevant cut-off values established to define cirrhosis in cases with viral or metabolic etiology cannot be applied. Chemello et al. demonstrated in a study involving 52 Fontan patients that LS values were significantly increased in cases with advanced FALD at cut-off values higher than 22 kPa (43). In the future, specialized cut-off values obtained by TE to identify the progression of FALD or advanced FALD have to be validated in large studies. To obtain this, standardizing liver stiffness ranges for congestion and fibrosis in the Fontan population may be useful. In addition, repeated TE testing might also be clinically useful in individual patients as a method to monitor their individual disease progression, indicate a hemodynamic deterioration, or recognize patients with a higher risk of congestive hepatopathy.

### 4.7. Clinical use in Germany

Our study shows that despite the given scientific background, TE is used in Germany only in a limited number (20%) of centers/hospitals specialized in Fontan follow-up. An additional 20% refer the patients to other departments and 20% to other centers. In 40% of the centers TE is however not used at all or even proposed (38.5%) in the Fontan Follow-up; in addition, the number of centers planning the implementation of TE is limited (3.2%). The Ultrasound equipment and software can be installed in all routinely available Echocardiography machines. The diagnostic assessment is quick to learn, easy to perform, not time consuming, simple, and very reliable and can be used during the routine echocardiography assessment during follow-up. Only a few centers/hospitals in Germany directly provided TE (20%) even though it is an ultrasound modality that can be implemented without specialized training. In only 2.6% of the centers is TE directly performed by the cardiologist. In 23.6% of the centers/hospitals, the patient is transferred to a pediatric radiologic department, in 7.9% to an adult radiologic department, or in 7.9% to another pediatric center. This causes additional time for the patients and families and may reduce the overall acceptance. Routine implementation in the echocardiographic workup of Fontan patients will certainly increase the widespread use of this important liver assessment tool.

### 4.8. Timing and indications for TE

To date, a standardized protocol on when to use TE, such as the starting time (age) of examination, the intervals or indications, does apparently not exist. Even though TE is easy to perform and possible without significant time delay, in most centers, the evaluation of TE is performed only when there is a given indication; in 28% (18 centers out of 95), TE was performed only for a given clinical indication. On the other side, in only 13.9% of the centers is TE performed in all the Fontan patients. Only a few centers perform TE in younger patients (i.e., 12.5% at school age and 15.6% before adolescence). In those centers/hospitals, where TE is routinely performed, it was mainly used in patients from 13 years of age (i.e., puberty) and older, and the follow-up intervals were 1–2 years (28%). It is however advisable to use TE before puberty to assess the trends of the individual patients and the potential changes during puberty and adolescence.

The technical recommendations published so far to perform reproducible results of TE include specific respiratory phases and breath hold maneuvers as well as fasting—both factors which might complicate a routine use during outpatient visits, especially for younger patients. These recommendations were however not known for the majority of centers (about 90%, see Table 4) and therefore do not seem to be important confounders in the use of routine TE. In addition, recent studies showed that for Fontan patients, the status of nutrition (whether they are fasting or not) as well as breath holding maneuvers do not influence the results of TE.

### 4.9. Future directions

Based on the published results it is evident that TE remains a practical and easy method to distinguish between cases with and without progressive FALD. Therefore, the liver stiffness follow-up is a useful and prognostic tool to monitor the clinical signs of failing Fontan circulation and Fontan-associated liver disease. We, therefore, propose to introduce TE systematically for the evaluation of liver congestion and fibrosis as a part of the routine follow-up of Fontan patients. The technical requirements can easily be implemented in the echocardiographic machines and without major costs. Even patients without clinical signs and symptoms of chronic liver disease should routinely undergo TE evaluation as part of their regular follow-up. Based on the known time characteristics of FALD, TE screening should begin at an age of 6 years and be conducted every 2 years. For adolescents and those older, it should be conducted yearly. Additional laboratory examinations with liver enzymes, platelet counts, liver synthesis parameters (protein, albumin, cholinesterase, coagulation) as well as stool examinations for protein-losing enteropathy (PLE, alpha-1-antiotrypsin) should be performed accordingly. It is important to monitor the TE (i.e., absolute liver stiffness value) over the time to identify the patients with progressive fibrosis and/or cirrhosis (27, 31) since TE will give an important additional reference value of liver stiffness for the individual patient. When introduced into routine follow-up in Fontan patients, not only the absolute values but more importantly the trends of the individual patients can be assessed over time. In addition, significant changes in TE values may be a good indication of changes in hemodynamics (i.e., stenoses, etc.). Significant changes in liver stiffness are observed when there is an increase of 20% of the liver stiffness over time or when absolute liver stiffness values have reached above 30 kPa (27). In those cases, in agreement with Schleiger et al., we suggest cardiac catheterization to assess hemodynamics, identify possible obstructions, modify the Fontan circulation, and improve hepatic venous outflow. A transvenous liver biopsy to stage the liver disease may be performed during the same procedure (27). Therefore, implementing TE in routine Fontan follow-up will improve the long-term management of Fontan patients.

## 5. Conclusion

TE is only used in a limited number of centers treating Fontan patients in Germany. A standardized follow-up protocol to detect FALD does not exist. Transient elastography is however a safe and highly accurate technique, easy to use, and well tolerated by the patients. It can be an effective monitoring and screening tool to identify patients with Fontan circulation affected by FALD and to stage those with a progress of FALD. We strongly suggest introducing TE systematically for the evaluation of liver congestion and fibrosis, as a part of the routine follow-up of Fontan patients, every 2 years, starting at the age of 6 years and on a yearly basis for adolescents and older. It should be an important part of a multi-modality approach to monitor Fontan associated liver disease. Implementing TE in routine Fontan Follow-up will improve the long-term management of Fontan patients.

## Data Availability

The original contributions presented in the study are included in the article/Supplementary Material, further inquiries can be directed to the corresponding author.
